# The Exceptional Child in Ontario

**Published:** 1919-08-16

**Authors:** 


### THE EXCEPTIONAL CHILD IN ONTARIO.

By A SOCIAL WORKER.

Except to those personally concerned, school reports are not generally interesting reading. But one which has come to us from Ontario has much in it which would appeal to all lovers of children, and especially at this moment of changes in our own educational schemes.

In 1914 an Act of the Provincial Legislature recognised a number of *auxiliary classes* to meet the needs of exceptional children, and in November 1917 the first grant-in-aid was paid to thirty-five of these, which had been organised according to the prescribed forms. Educational experiment for the *hors-ligne* cases has thus been encouraged as Mr. Fisher's Act encourages it in our own country, especially in the nursery school. In all countries it is being realised that the child is not a mere lump of paste to be moulded similarly to a number of other buttons.

The present categories of recognised special classes in Ontario are, first, an advancement class for the child mentally and physically over average—a wholesome remedy for the "slacking" and conceit that are easily fostered in a young mind that is kept back among inferiors; at the other end of the scale a promotion class for the backward who is not actually defective; an "English" class for foreign child-immigrants, to include adults if desirable, and special disciplinary classes or parental schools for children of neglected homes or difficult characters. In the Province of Ontario two such schools for girls and two for boys are at work, most of the pupils having been committed by the courts. It is not surprising to hear that a very large proportion of these are mentally defective, and need to be passed on to special institutions. But in our own country there is need for attention to that incipient wastrel or criminal, the slum child whose mother states it as a simple fact that she "can't do nothink with him" at four and a-half or so, and warns the teacher when he enters the infant school that she may lead but cannot hope to drive him. Some special experience of the control that shall lead to self-control would seem to be wanted.

### Physically Handicapped Children.

But now for the sick, the delicate, the physically handicapped children. In England during the influenza epidemic doctors appear to have spent most of the time of a school visit in opening more and more windows, and here and there we have open-air schools for tuberculous children in summer. Ontario recognises open-air work not merely for recognised consumptives, but for "delicate, anæmic, or under-nourished children, held in forests, parks, or fields, or in class-rooms, one side of which at least is open to the sun and outer air." Orde Street School, in Toronto, has started two such class-rooms, one warmed by radiators, the other not at all, but this is condemned as too severe. We in England must certainly reconsider our habit of keeping ourselves and the children in "paper-lined boxes" during so much of our lifetime. The habit of pursuing ordinary home avocations, especially such sedentary ones as needlework, is one which Englishwomen need to cultivate, especially perhaps those of the lower middle class who coop themselves and their children up in small rooms, except during very limited times of formal outing. Our eager house-planners will be wise if they study the contriving of paved courts and sunny terraces of brick, tile, or gravel where chairs and tables could stand, with a bit of roof against rain and of awning for summer sunshine.

Toronto authorities were much encouraged by the success of a scheme in Iowa where a school of forty hospital children was started in November 1916. Tests made then and in the Christmas holidays revealed the remarkable fact that practically a year's ordinary progress had been made. Among institutions where classes are held, shelters are mentioned, a valuable suggestion for efforts that might be made with advantage here among children who for a time are best kept apart from others, and who need even more than others the benefit of wholesome occupation and interest in the best things of life. Another useful hint for us is the interest taken by members of the Ontario Women's Institutes in medical inspection of rural schools. Mr. Cyril Leeson, secretary of the Howard Association, some time ago suggested the establishment of local councils for the meeting of children's war-time needs. We do not want to multiply organisations, and without this it should be possible to induce the spirit of mothering that begins at home but does not end there.

### Classes for Epileptics.

Special classes for epileptics are recognised, and as in all hospital classes individual teaching is admitted when necessary, we may hope that these afflicted little ones will not be allowed to aggravate each other's cases. Three other important sections are classes for children so myopic that not even a front seat and spectacles enable them to profit by ordinary teaching, in lip-reading for those so deaf that they may lose all hearing, and for stammerers. The formation of such classes naturally leads to preventive measures. As regards eyesight, much can, of course, be done to prevent damage by measures against ophthalmia neonatorum, by provision of proper desks, lighting, and type in school, and prescription by oculists, besides protection alike from too much study and too much picture-palace attendance But the slum and city street appear in themselves to cause myopia. The eye that never needs to see further than across the narrow street lacks training. For the deaf, attention is naturally drawn to the cure of adenoids and of bad mouth conditions, and it is hoped in Ontario as here that efforts now made to restore hearing to soldiers who have suffered from high explosives and shell-shock will react upon the little ones. It is very interesting to learn that an evening class for young women at Toronto has proved so acceptable that attendances have averaged nearly 100 per cent.

It follows naturally from the statement of effort and experience here sketched that teachers suited to special classes are needed in increasing numbers. The same will be the case in our own country, of course, and opportunity of new and much-needed service will open to girls who have done V.A.D. work but do not wish to make nursing their life-work. Teachers of the "retarded" child are felt to be needed in Ontario, and natural gifts of sympathy and discernment will be cultivated by some special training. And so with regard to the child deficient in sight and hearing, and the disabled. Let no one ask despairingly, "What on earth will these young women do now?" "His reward is with Him and His work is before Him" Who works through human agents.

